# Uncontrolled admixture and loss of genetic diversity in a local Vietnamese pig breed

**DOI:** 10.1002/ece3.229

**Published:** 2012-05

**Authors:** Cécile Berthouly-Salazar, Sophie Thévenon, Thu Nhu Van, Binh Trong Nguyen, Lan Doan Pham, Cuong Vu Chi, Jean-Charles Maillard

**Affiliations:** 1 Department of Botany & Zoology, DST-NRF Centre of Excellence for Invasion Biology (C·I·B), University of StellenboschMatieland 7602, South Africa; 2 Centre de Coopération Internationale en Recherche Agronomique pour le Développement (CIRAD)UMR INTERTRYP, 34398 Montpellier, France; 3 National Institute of Animal Husbandry (NIAH)Tu Liem, Hanoi, Vietnam; 4 Centre de Coopération Internationale en Recherche Agronomique pour le Développement (CIRAD)UPR AGIRs, 34398 Montpellier, France

**Keywords:** Admixture, crossbreed, exotic, hybrids, pig, Vietnam

## Abstract

The expansion of intensive livestock production systems in developing countries has increased the introduction of highly productive exotic breeds facilitating indiscriminate crossbreeding with local breeds. In this study, we set out to investigate the genetic status of the Vietnamese Black H’mong pig breed by evaluating (1) genetic diversity and (2) introgression from exotic breeds. Two exotic breeds, namely Landrace and Yorkshire used for crossbreeding, and the H’mong pig population from Ha Giang (HG) province were investigated using microsatellite markers. Within the province, three phenotypes were observed: a White, a Spotted and a Black phenotype. Genetic differentiation between phenotypes was low (0.5–6.1%). The White phenotypes showed intermediate admixture values between exotic breeds and the Black HG population (0.53), indicating a crossbreed status. Management practices were used to predict the rate of private diversity loss due to exotic gene introgressions. After 60 generations, 100% of Black private alleles will be lost. This loss is accelerated if the admixture rate is increased but can be slowed down if the mortality rate (e.g., recruitment rate) is decreased. Our study showed that a large number of markers are needed for accurately identifying hybrid classes for closely related populations. While our estimate of admixture still seems underestimated, genetic erosion can occur very fast even through indiscriminate crossbreeding.

## Introduction

Since the domestication of animal species, livestock movements and trade have been important driving factors for the genetic make-up of existing breeds, with selection and drift. One of the best examples is the high diversity of African cattle breeds, which results from different migration waves of taurine and zebu cattle (Hanotte et al. 2002). However, since the middle of the 20th century, technological advances have been facilitating gene flows throughout the world, increasing their rate and spread (Mathias and Mundy 2005; Hiemstra et al. 2006; Valle Zárate et al. 2006).

Toward the end of the 20th century, gene flow reached developing countries, resulting in an expansion of intensive livestock production systems; an expansion referred to as the “livestock revolution” (Delgado et al. 1999). Increasing demand has led to the use of a limited number of breeds for intensive production systems sidelining local breeds. In developing countries where small-scale farming systems remain the main source of production (Huynh et al. 2007), different strategies for increasing productivity have been implemented either via the direct use of improved exotic breeds or by crossbreeding. Despite the fact that there are well-documented examples of unsuccessful projects using exotic breeds (Sainath 1996; Ayalew et al. 2003; Rutledge et al. 2010), such a strategy is still common practice. Crossbreeding of local and exotic improved breeds can show a faster impact on performance than long selection schemes for the improvement of local breeds, and are therefore more beneficial in the short term. However, as another consequence, it is one of the major threats for the disappearance of local genetic diversity, inducing displacement (Hanotte et al. 2010) or genetic erosion. Indiscriminate crossbreeding or extensive use of exotic germplasms can lead to genetic erosion by dilution or eradication of the local genetic pool (Rege and Gibson 2003). Recent studies have been warning about the endangered status of animal genetic resources (Taberlet et al. 2008, 2011).

FAO (1999) identifies two main weaknesses in monitoring local domestic diversity in developing countries: (1) many nondescript local breeds for which it is unclear whether they form homogenous groups; and (2) the current monitoring of breed erosion does not capture genetic dilution of local breeds by indiscriminate crossbreeding. Few reports of genetic erosion are found in the literature for local breeds in developing countries, merely some examples on Ankole cattle in Africa, Vechur in India, Kao Lumpoon cattle in Laos, Namaqua and Red maassai sheep in Africa (Katushabe et al. 2001; Mathias and Mundy 2005 and references therein). However, to our knowledge, there is no study to date that clearly investigates genetic erosion by admixture of exotic breeds in local breeds. In this study, we set out to address the main two constraints encountered by FAO in monitoring local diversity: (1) the genetic characterization of the local breed, namely the Vietnamese H’mong breed, and (2) the estimation of its genetic dilution.

In Vietnam, pig production is divided into smallholder production and small-medium, medium, and large production. Medium and large production involves more than 20 sows and use of exotic breeds. These farming systems are centralized in areas of high population density and amount to 10% of the Vietnamese pig population (Gauthier et al. 2009). Smallholder systems dominate pig production in Vietnam (Thong et al. 1996) and account for about 80% of the total pig population (Kinh et al. 2002). Imported exotic breeds in Vietnam include Landrace, Yorkshire, Duroc, Hampshire, and Pietrain (Gauthier et al. 2009). According to the Vietnamese Ministry of Agriculture and Rural Development (MARD), current research investment priorities for genetics are to develop large-scale commercial/industrial pig production in concentrated areas (the Red River, Mekong delta, and the Southeast zones), and to create a nucleus herd of pure exotic breeds and crossbred breeds (Kinh et al. 2002). One of the most common crossbreeds uses the Mong Cai breed, as its utility for fattening in a cross with exotic pig breeds has been demonstrated (Rodríguez and Preston 1997). Yorkshire and Landrace breeds are commonly used in crossbreed programes, as with the Mong Cai breed in northern provinces (Le and Tuyen 1998; Huong Tra 2003). According to the Vietnamese authorities, there is no official importing of animals from neighboring countries, but unofficial practices have been observed. The only pigs that are moved from China to Vietnam are local pigs traded between villages on both sides of the border (Cocks et al. 2009). In Vietnam, the percentage of indigenous sows declined from 72% of the total population in 1994 to only 26% in 2002 (Huyen et al. 2006). Of its 14 local breeds, five are now considered as vulnerable, two are in a critical state, and three are facing extinction. One of the most emblematic Vietnamese local breeds is raised by the H’Mong ethnic group inhabiting the northern Ha Giang (HG) province bordering on China. They raise the H’mong black chicken, which is considered to have medicinal properties (Berthouly et al. 2009a), H’mong cattle, which are one of the largest Vietnamese breeds (Berthouly et al. 2010), and finally the H’mong black pig. According to consumers and farmers, this pig breed, which is assumed to have a Black coat, produces more and better fat than other breeds. Although its cultural value is high, little is known about its genetic status. Geographical characteristics and farming practices may have structured this pig population that has been isolated for decades. However, since the 1980s with the Doi Moi policy, HG province has recently been reached by the “livestock revolution,” involving productivity and exotic breed introductions. In northern provinces, pigs resembling exotic white breeds have been imported, mainly from China. They also show similar characteristics, such as higher food demand, but they are already vaccinated. In a province where householders lose their animals to diseases every year, this is a valuable point. Migrations from neighboring provinces and governmental policies have led to industrial farms using exotic breeds, namely Landrace and Yorkshire and their crossbreed (Gauthier et al. 2009). In HG city, the capital of the province, “intensive” farms with 10–30 pigs have been created (i.e., the biggest farms in that province). HG householders have therefore started using “exotic like” pigs from China or from intensive farms.

Surrounded by exotic pigs, the H’mong black pig may not resist and little is yet known about it. What is its genetic pattern? Is it already crossbred? What is the effect of indiscriminate crossbreeding on its genetic pool? How long will it take to lose its specific genetic pool if no conservation programes are launched? These are the main questions that this study focused on. Genetic data, phenotypic data, and management practices were combined to estimate the degree of introgression to date and to predict the effects of a prolonged introgression.

## Materials and Methods

### Sampling procedure

**Figure 1:**
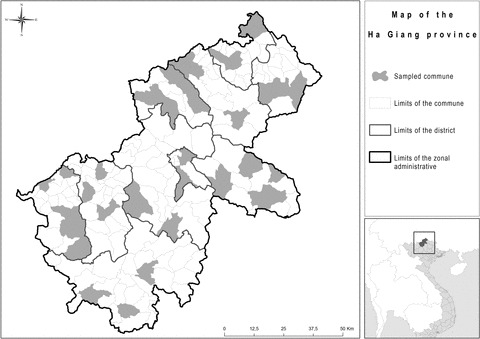
Map of Ha Giang (HG) province. (A) District borders (bold lines); (B) sampled commune in pale gray.

The remote Vietnamese province of HG (Fig. 1, 22°08′–23°19′N; 104°33′–105°33′E) is split into districts (11), communes (193), and villages (1715). A stratified sampling protocol was applied, with three strata: district/commune/village. The 11 districts were surveyed with one to five communes per district (35 in total) and one to eight villages per commune (207 in total). On average, three unrelated pigs per village or 16.3 pigs per commune were sampled. For each of the 567 samples, geographic coordinates, measurements, and genotypes were recorded in a database linking morphometric information to molecular information. The commune was the scale on which *F*-statistics were computed. According to the National Institute of Animal Husbandry (NIAH), improvement programes have been implemented in northern provinces using two exotic breeds: Landrace and Yorkshire. Samples from these breeds were collected at the NIAH in Hanoi: 22 and 25 individuals for Landrace and Yorkshire, respectively.

### Microsatellite markers

Genomic DNA was extracted from tissue samples using the QIAamp Kit from QIAGEN. The PCR products were labeled with fluorescent dyes and genotyped using a capillary sequencer (Beckman Coulter CEQ8000) for 16 microsatellite markers recommended by FAO (S0026, S00355, S0068, S0097, S0155, S0215, S00226, S0227, SW1067, SW122, SW240, SW2410, SW632, SW72, SW857, SW936). These markers belonged to different chromosomes, except SW1067 and SW122, both mapping to chromosome 6. Genotypes from this study are available on request.

### Genetic analyses

#### Summary statistics

The presence of null alleles was tested using FreeNA (Chapuis and Estoup 2007): loci with estimated frequencies of null alleles *r*≥ 0.2 were considered to be potentially problematic for calculations. Expected and Observed heterozygosities were calculated using GENETIX 4.4 (Belkhir et al. 2000). GENEPOP software (Rousset and Raymond 1995) was used to compute *F*-statistics (Weir and Cockerham 1984) and Hardy–Weinberg equilibrium tests (Rousset and Raymond 1995). Test significance was corrected with sequential Bonferroni correction on loci. Allelic richness and private allelic richness were calculated using the rarefaction method of HP-Rare software (Kalinowski 2005). During the surveys, three phenotypes were observed: (1) a white phenotype with some black skin spots and with straight ears looking similar to the Yorkshire breed, called White pig in the rest of the text (49 samples); (2) a black spotted white-haired phenotype with hanging ears (252 samples) called Spotted pig; (3) a black phenotype with hanging ears corresponding to the Black H’mong pig breed (266 samples) called Black pig. In this study, we used “White” pig for white pigs from HG province, and “Exotic” pig for the Landrace and Yorkshire pigs sampled. An earlier morphometric study (Berthouly et al. 2007) showed that body traits could be used to differentiate between the White and the Black HG populations, but not between the Black and the Spotted HG populations. White HG pigs have long and vertical ears that are also characteristic of the Yorkshire breed, whereas Black and Spotted HG pigs are characterized by long hair, hanging ears, and a belly touching the ground.

#### Genetic structure identification

We investigated the genetic structure and individual assignments using a Bayesian clustering procedure implemented in STRUCTURE (Pritchard et al. 2000), with the admixture model and correlated allele frequency (Falush et al. 2003) from *K*= 1–10, 10 times each with 10^6^ iterations following a burn-in period of 300,000. The values for the number of clusters (*K*) were assessed according to Evanno et al. (2005) by the (*D*·Δ*K*) criterion after testing for *K*= 1 using the Pritchard approach (Pritchard et al. 2000). The program estimated the posterior distribution (*q*) of each individual's admixture coefficient. STRUCTURE software was run first for the exotic and HG dataset (three HG phenotypes, Landrace and Yorkshire breeds), then for the HG dataset (i.e., the three phenotypes), and finally for the Black population only.

We tested the genetic pattern of the Black HG phenotype for isolation by distance (IBD) based on pairwise kinship coefficients between individuals using SPAGeDi 1.3 software (Hardy and Vekemans 2002). As suggested by Vekemans and Hardy (2002), the kinship coefficient presented in Loiselle et al. (1995) was chosen as the pairwise estimator of genetic relatedness. Kinship coefficient values (*F_ij_*) were regressed on *d_ij_*, where *d_ij_* was the spatial distance between individuals *i* and *j*. Standard errors were assessed by jackknifing data for each locus. Spatial correlation between *F_ij_* and *d_ij_* was tested by permutation (9999) on individuals. Significant correlations were associated with the IBD process.

#### Hybrid discrimination

In order to assess the discrimination power of our micro-satellite set, we generated 100 individuals for each of four genotypic classes that originated after two generations of hybridization, that is, first-generation hybrids (F1), second-generation hybrids (F2), backcrosses of F1 with the first parental population (BE), backcrosses of F1 with the second parental population (BL). Parental source populations were the Exotic (Landrace and Yorkshire mixed) breed and the local Black HG population. Genotypes were generated using HYBRIDLAB software (Nielsen et al. 2006).

We then used NEWHYBRIDS software (Anderson and Thompson 2002) to compute the posterior probability (*q*′) of each individual belonging to each of six genotypic classes that originated after two generations of hybridization as described above. We ran the program for two datasets: (1) the simulated dataset with Exotic and Black parental populations and (2) the real datasets with Exotic (P0), Black (P1), Spotted, and White pigs. For both runs, we provide a priori population information for all the Exotic samples and for 10 individuals from the Black HG population. The Black HG individuals were selected as the ones showing the highest *q*-values from STRUCTURE analyses. We used a burn-in period of 150,000 followed by 500,000 iterations. The simulated dataset was also then evaluated using STRUCTURE in a similar manner to the empirical data from *K*= 1 to *K*= 7.

### Modeling of management practices and future evolution of allelic frequencies

Householders were questioned about their management practices. They were asked to estimate the age at first parturition, the farrow interval, and the mortality rate (MR). According to the interviews, the householders did not practice any selection for reproduction. Reproductive sows were raised freely, while most of the fattening pigs were shut in sheds. However, personal observations and interviews revealed that they often escaped. This information, plus the admixture rate (AR) estimated from genetic data, was used to create a model designed to estimate the evolution of allelic frequencies and admixture in the Black population. The model was run using R software (R Core Development Team 2006) and made it possible to estimate the evolution of allele frequencies in the local population in view of management practices. The parameters used in the model were allele frequencies, the AR, and the MR. According to the surveys, 9% of the pigs recorded were White pigs. However, that proportion was underestimated because, at the time, we were focusing on sampling local pigs; therefore, our genetic estimate of AR was used instead in the model. We considered that the parental stock was composed of 1000 reproductive pigs. For generation 0, two parental stocks were used: B0 (Black population) and W0 (White population), both using allele frequencies from this study. At generation B1, a pool of 2000 gametes was constituted of (1 – AR) gametes randomly selected from B0, and AR gametes from W0. From generation *n*+ 1, because an overlapping generation existed, the new generation participated in the reproductive stock at the mortality rate (MR): MR × (1 – AR), (1 – MR) × (1 – AR) gametes from parental population B*n*– 1 were randomly selected, and AR gametes from W0 stock, which remained constant as these pigs were bought from outside and only kept for one year. Our model considered a constant population size and took into account genetic drift by randomly selected gametes from the gamete pool. The parameters were one batch of 1000 individuals for 100 generations in a constant population size. The model was replicated 100 times. We tested three values for the AR, AR = 0 as no admixture, AR = 0.25, as estimated by us, and AR = 0.5, and two for the MR, MR = 0.4, following our interviews, and MR = 0.2. In the following analysis, an allele was considered as lost when its frequency was equal to 0.

## Results

### The Black, the White, and the Spotted phenotypes

The expected heterozygosity was similar for all three phenotypes (0.791–0.804) (Table 1). Regarding the observed heterozygosity, the Black HG population showed the lowest value (0.596), followed by the Spotted (0.605), and the White population showed the highest value (0.742). The Black and Spotted populations had 15 alleles that were not found in the White population and 83 alleles not found in the two Exotic breeds. When using a rarefaction method based on the smallest sample size, the average numbers of private alleles per locus were 0.39 for the Exotic population, 0.13 for the White population, and 0.31 for the Black population. The *F*_IS_ values were at least four times higher for the Black and Spotted populations (0.26 and 0.25, respectively) than for the White population. The highest genetic differentiation was found between Landrace and Black HG populations (18%). Genetic differentiation between the White and Black HG populations was 6.1% and 4.1% between the White and Spotted HG populations. The *F*_ST_ value was low (0.5%) between the Black and Spotted HG populations.

**Table 1:** Summary of genetic statistics.

Per population	*N_i_*	*H*_e_	*H*_o_	Mean number of alleles per locus	*F*_IS_
Landrace	22	0.632	0.617	5.8 (5.62)	0.024
Yorkshire	25	0.606	0.555	5.4 (5.17)	0.085
White HG	49	0.791	0.742	9.1 (7.84)	0.063
Black HG	266	0.803	0.596	10.0 (8.36)	0.258
Spotted HG	252	0.804	0.605	10.1 (8.59)	0.248

*N_i_*, number of individuals; *H*_e_, unbiased expected heterozigosity; *H*_o_, observed heterozigosity; *F*_IS_, inbreeding coefficient. Mean number of alleles per locus using rarefaction methods are shown in parentheses.

**Figure 2:**
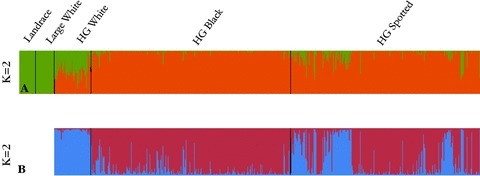
Summary of admixture results. (A) Using Exotic breeds and HG populations; (B) using HG populations (White, Spotted, and Black).

**Figure 3:**
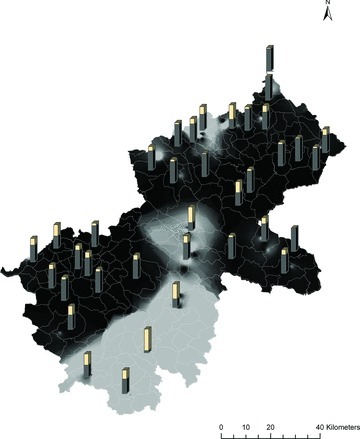
White phenotype distribution (grid map) and mean *q*_1_ values for Exotic cluster (bars). The background color represents the probability of observing white phenotypes. The lighter it is, the higher is the proportion of white phenotypes. Bars represent the average *q*_1_ value per commune of belonging to the Exotic cluster.

When we performed the Bayesian procedure on the global dataset (i.e., the three phenotypes and the two exotic breeds), we obtained a subdivision into two clusters. (*D*·Δ*K* was the highest for *K*= 2; Fig. S1.) The two exotic breeds, Landrace and Yorkshire, belonged to cluster 1 with *q*_1_ probability >0.99 (Fig. 2A). The Black HG population belonged to cluster 2 with a mean *q_2_* probability of 0.97 ± 0.05 SD and the Spotted HG population with *q_2_*= 0.92 ± 0.14 SD. The White HG population was a mixture of both clusters with *q_2_*= 0.53 ± 0.18 SD (Fig. 2A). Histograms of *q*_1_ values averaged per commune plotted on a grid map of phenotype frequencies are shown in Figure 3. The high proportion of white phenotypes observed per commune was congruent with the membership values of the white cluster.

When analyzing the HG population alone, we found *K*= 2 with one cluster represented by the White population (*C_W_*) and the other by the Black population (*C_B_*) (Fig. S1). Within the Spotted population, individuals seemed to belong either to the white cluster (22% with *q_CB_* > 0.9) or the black cluster (49% with *q_CW_* > 0.9). Only 10% of Spotted pigs showed intermediate values (0.3 < *q* < 0.7). The *q_CB_* values averaged across individuals with Black and Spotted phenotypes reached a mean AR of 0.25 with the White phenotype cluster. Following our results, we put forward the hypothesis that the White phenotype is the result of an admixture between exotic and local breeds, and the Spotted phenotype is the result of multigeneration crossing.

The Bayesian procedure on Black HG pigs revealed no genetic subpopulations. The value of the criterion used was the highest for *K*= 1 following Pritchard's procedure. The values of the kinship coefficient averaged over a set of distance classes were plotted against geographical distance (Fig. S2). Considering all pairs of individuals, the regression slope *b*± SE = 0.0097 ± 0.0018 was highly significant (*P* < 0.00001). Individual kinship coefficients negatively correlated with geographical distance showing a logarithmic relationship, indicating an IBD process.

### Hybridization and accuracy of hybrid discrimination

**Figure 4:**
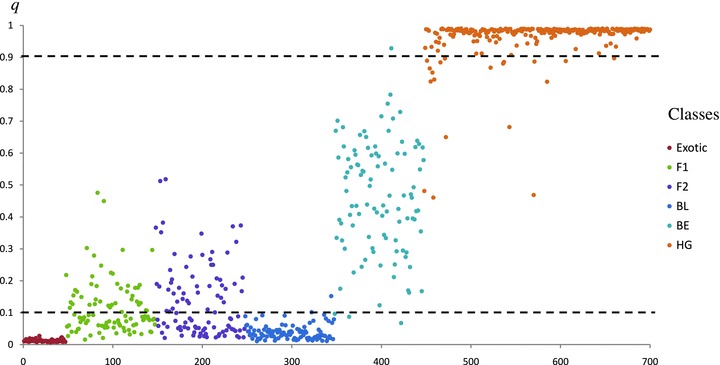
Estimates of *q*-values using STRUCTURE for simulated genotypes of each of the six possible “genotypic classes” of parentals and hybrids that are created accounting for two generations of mating between Exotic (cluster 1) and Black HG (cluster 2) populations. Dotted lines indicate *q*_1_= 0.1 and *q*_2_= 0.9.

We first ran STRUCTURE on the simulated dataset with Exotic and Black HG as parental populations. We found that *K*= 2, thereby representing the two parental clusters, Exotic (E) and Black (B). As such, we expected that parental (i.e., pure) individuals would show *q*-values > 0.9 and all hybrid classes should have *q*-values < 0.9 and near to 0.5, at least for F1 and F2. The results showed that 92% of HG Black could be identified as parental individuals (*q_B_* > 0.9; Fig. 4). Conversely, differentiation was not possible, at least between exotic pure and exotic backcross pigs, with 100% of exotic parental individuals and 98% of exotic backcrosses showing *q_E_* values > 0.9. Both F1 and F2 showed *q*-values lower than 0.4, which did not allow their distinction. Backcrosses with Black HG had the most intermediate *q-*values ranging from 0.09 to 0.92.

While STRUCTURE is used to estimate ARs, NEWHYBRIDS is assumed to distinguish first- or second-generation crosses more accurately. Using NEWHYBRIDS on the simulated dataset, the efficiency of our marker set in discriminating between crossbreeding classes was very variable (Table 2). More than 92% of individuals for both parental populations (at a threshold *q*′ > 0.7) were assigned as parents. In addition, no hybrid of any class was assigned as a parent. Of the individuals from class F1, 55% were assigned correctly and 11% missassigned to other hybrid classes. F2 individuals showed a substantially higher rate of assignment at *q*′ > 0.9 compared to other hybrid classes. This difference was attenuated when considering an assignment threshold of *q*′ > 0.7. The lowest correct rate (15%), at *q*′ > 0.9, was found for Black HG backcrosses (BL). In addition, they also showed the lowest similar missassignment rate (13%). Of the individuals assigned to F1 and F2 classes, 83% and 80%, respectively, were true F1 and F2 for *q*′ > 0.7.

**Table 2:** Percentage of individuals assigned to a class using the simulated dataset by HYBRIDLAB.

			Assigned to					
*q*′ threshold	True class	*N_i_*	Exotic	Black	F1	F2	BE	BL
*q’* > 0.9	Exotic	47	83					
	Black	266		93		1		
	F1	100			30	3	2	
	F2	100			2	45	4	
	BE	100			1	2	25	
	BL	100			1	13		15
								
*q’* > 0.7	Exotic	47	92					
	Black	266		95		1		1
	F1	100			55	6	5	0
	F2	100			4	58	8	
	BE	100			5	8	52	0
	BL	100			2	25		42

*q*′ is the posterior probability obtained from NEWHYBRID analysis; results are presented for *q*′ > 0.9 and *q*′´ > 0.7. *N_i_*, number of individuals tested; BE, backcrosses with Exotic pigs; BL, backcrosses with Black Ha Giang pigs.

NEWHYBRIDS run on the empirical data identified more than 98% of Black HG as phenotypic Black HG and 100% of Exotic pigs as Exotic ones (Table 3). According to this analysis, 86% of Spotted HG individuals were classified as pure Black and 7.5% as crossbreds. A total of 59% of White HG pigs fell into the F2 category and, surprisingly, 6% into Black HG. Conversely, this means that 35% of White HG pigs showed *q*′ values below 0.7 and were thus not assigned to any category.

**Table 3:** Assignation results of the Exotic and Local HG pigs dataset.

			Number of assigned individuals per *q*′ range			
Phenotypic class	Number of genotyped individuals per class	Assignation group	*q*′ > 0.9	*q*′: 0.8–0.9	*q*′: 0.7–0.8	Total individuals assigned with *q*′ > 0.70 and (%)
Exotic	47	Exotic	47	0	0	47 (100)
HG Black	266	HG Black	257	1	4	262 (98)
		F2	1	0	0	1 (0.4)
HG Spotted	252	HG Black	206	7	3	216 (86)
		F2	6	6	2	14 (06)
		HG Black-F1	1	3	1	5 (02)
HG White	49	HG Black	2	0	1	3 (06)
		F2	20	4	5	29 (59)

*q*′ is the posterior probability obtained from NEWHYBRID analysis.

### Effect of introgression on the evolution of private alleles

According to the householder interviews, the average age of first parturition was at 18 months and the farrow interval was about 8.5 months. On average, each sow was used for two parturitions, and the natural MR in the population averaged 0.4. Mortality was used as a recruitment rate for the reproductive stock in the model, leading to a constant population size (1000 reproductive individuals), the remaining pigs being sold as meat carcasses.

We first tested our model with no admixture and only genetic drift. After 100 generations, 17% of alleles were lost. Among alleles lost, 38% were from exotic origin from previous admixture. Only four of the 15 privates Black alleles were lost.

According to the householders, White pigs were raised for less than one year and only for fattening. In other words, each year the householder bought new White pigs. Consequently, the allele frequencies of the White pigs were assumed to remain constant over the years. The householders did not state that they used White pigs for reproduction but escapes and uncontrolled reproduction can happen. We therefore considered that the probability of crossbreeding was equal to the mean AR (*q_CB_*= 0.25 averaged across Black and Spotted phenotypes; see above). This frequency was considered constant across generations; in other words, reproduction with White pigs always amounted to 25% in the HG pig population.

We ran the model for 100 generations and for each locus. At time 0, the Black phenotype population showed 15 privates alleles. According to the model, after 30 generations, 47% of private alleles were lost. At 50 and 60 generations, the percentage of lost alleles reached 93% and 100%, respectively. Of the 15 Black private alleles, 15 were lost and two new, previously private alleles from the White population, were gained. In total, 10% of alleles were lost. Expected heterozygosity passed from 0.79 to 0.78 and effective alleles from 5.3 to 4.9 after 100 generations (Table 4). While at *t*= 0, *F*_ST_ between White and Black HG was 0.061, after 100 generations of crossbreeding *F*_ST_ would be 0.0002.

**Table 4:** Evolution of allele loss per locus: generation times at which an allele is lost.

	Generation times								
	Admixture (0.5)		Admixture (0.25)						
Mortality	0.4	0.2	0.4	0.2	Percentage of allele lost	*H*_ei_	*H*_ef_	*A*_ei_	*A*_ef_
Sw2410					0	0.85	0.79	7.0	4.5
SW240					0	0.71	0.7	3.5	3.3
S00355	14	20	33	45	9	0.81	0.81	5.4	5.3
SW1067					0	0.84	0.82	6.3	5.7
S0226	10		21	39	8.3	0.77	0.70	4.4	3.3
		17	26	49	16.6				
	20	21	44	53	25				
S0215	2	4	10	7	8.3	0.82	0.68	5.5	3.2
	10	12	36	30	16.6				
	25	14	40	59	25				
S0227	8	11	18	21	10	0.82	0.62	5.5	2.7
	18	30	21	29	20				
S0026	18	25	26	80	12.5	0.79	0.75	4.9	4.1
S0155	18	41	38	33	10	0.69	0.80	3.2	5.2
Sw632					0	0.76	0.85	4.1	6.5
Sw936	5	6	22	3	7.7	0.84	0.83	6.4	6.0
S0068					0	0.81	0.89	8.0	8.8
Sw122					0	0.63	0.75	2.7	4.0
Sw857	11	30	16	36	9	0.86	0.81	7.1	5.1
	20	58	45	70	18.2				
S0097	16	23	53	76	10	0.83	0.82	5.9	5.3
Sw72					0	0.81	0.82	5.4	5.6

Range	2–25	4–58	10–53	3–80	0–53				
Average					0.79	0.78	5.3	4.9	

Expected heterozygosity at *t*= 0 (*H*_ei_) and *t*= 100 (*H*_ef_); Effective number of alleles *t*= 0 (*A*_ei_) and *t*= 100 (*A*_ef_).

We then tested the model using a higher AR and a lower MR. The model with the highest admixture and MRs showed the highest acceleration in allele loss. After 10 generations, 33% of private alleles were lost and 100% after only 30 generations. The admixture effect was greater on genetic erosion compared to the MR effect. When the AR was increased from 0.25 to 0.5, it accelerated by 13.7 generations, on average, while when the MR was increased from 0.2 to 0.4, it accelerated by 9.7 generations, on average.

## Discussion

Our findings demonstrate that introgression involving exotic breeds makes a substantial contribution to the genotypes of the Vietnamese H’mong pig breed. Although introgression between local and exotic breeds has rarely been studied in developing countries, the situation discovered seemed similar to introgression described for domestic–wild species, or wild–wild species, with consequent genetic swamping and extinction of locally restricted gene pools (Frankham et al. 2002; Berthouly et al. 2009a; Chazara et al. 2009; Hertwig 2009; Cabria et al. 2011). Using microsatellite markers, we were able (1) to characterize the genetic pattern of the H’mong pig breed; (2) to show that crossbreeding has occurred and involved many generations of backcrossing; and (3) that indiscriminate crossbreeding will rapidly lead to genetic erosion of the local H’mong pig breed.

### Characterization of the Vietnamese local H’mong pig breed

According to FAO (1999), a major problem for the management of Animal Genetic Resources (AnGR) is that there are many local breeds for which it is unclear whether or not they form homogenous groups. For instance, Zimbabwe chickens seem to form a unique genetic pool (Muchadeyi et al. 2007). Rout et al. (2008) showed that Indian goat breeds could be classified in two major genetic clusters. Zhang et al. (2007) studied 27 indigenous Chinese cattle breeds. They highlighted that grouping breeds according to their geographical distribution was not always consistent because of human migratory movements. While HG cattle and goat populations are structured into subpopulations (Berthouly et al. 2009b, 2010), HG chicken and buffalo populations should be considered as one genetic pool (Berthouly et al. 2009a, c). Similarly to chickens and buffaloes, the Black H’mong pig population can be considered as one population showing no genetic subdivision. However, a very clear IBD pattern was found, associated with a high *F*_IS_ value. Such a pattern is in agreement with management practices and continuous distribution. Pigs are usually sold at commune level or between neighboring villages, simulating a stepping-stone diffusion model. From a conservation perspective, these results would have two consequences. First, implementation or facilitation of exchanges of reproductive animals at province level should be organized in order to decrease inbreeding. Second, national Vietnamese institutions might decide to create either an ex-situ or in-situ conservation herd; it would need to select a representative sample of the genetic diversity found in the province. In order to do so, they could also take into consideration a coloration criterion when selecting the first reproductive stock and only select the Black phenotype. In addition, a policy should be established to limit introgression mainly in the central and southern areas of the province. These areas showed the highest rates of introgression and frequencies of White pigs, probably due to the fact that main roads connecting China and southern Vietnamese provinces pass through these areas.

### Identification of hybrids

The development of molecular genetic markers has greatly benefited AnGR conservation issues. Microsatellite markers combined with modern analytical approaches for clustering (Manel et al. 2005) can tease apart the genetic relationships and uniqueness of breeds, making it possible to prioritize breeds for conservation (Caballero and Toro 2002; Reist-Marti et al. 2003). However, a crucial point for a conservation project is to estimate the “purity” of the breed and thus, to accurately document the degree of hybridization and introgression. To date, Bayesian softwares have generally proven to be efficient in identifying pure breeds (Vähä and Primmer 2006; Sanz et al. 2008; Marie et al. 2010). In this study, both admixture softwares were able to correctly identify Black HG breeds (at least 93%). By contrast, NEWHYBRIDS correctly identified Exotic pure breeds while STRUCTURE was not able to distinguish them from simulated exotic backcrosses. When it came to the detection of the subsequent generations of crossbreeding, the power was more limited. Discrimination power has been investigated by several authors. Vähä and Primmer (2006) showed that to efficiently identify F2 and backcrosses as well as F1, 48 loci with an average pairwise *F*_ST_= 0.21 would be necessary. Using nine microsatellites and an average *F*_ST_= 0.26, Sanz et al. (2008) were able to discriminate between parental populations and hybrids but not between hybrids. In our simulated dataset, NEWHYBRIDS, with 100% of hybrids identified as such, was more accurate than STRUCTURE (48%). Conversely, Marie et al. (2010) found that STRUCTURE was more accurate in discriminating F1 than NEWHYBRIDS (99.4% and 76%, respectively) but that STRUCTURE had a higher rate of missassigned backcrosses in parental classes than NEWHYBRIDS (30% and 10%, respectively). We found similar observations with Exotic backcrosses being assigned to pure Exotic by STRUCTURE, whereas NEWHYBRIDS backcrosses were missassigned within hybrid classes. Schwartz and Beheregaray (2008) studied introgression between Australian bass and perch populations. Unlike our results, in their study the first generation seemed to discriminate better than backcrosses. While in our cases, *F*_ST_ values reached a maximum of 0.18, *F*_ST_ values approximated to 0.7 in the case of Australian fishes. According to Marie et al. (2010), *F*_ST_ values may not be alone in influencing the accuracy of NEWHYBRIDS results. For instance, it performed better for hybrid discrimination when higher proportions of hybrids were found in the data. More than 40% will significantly improve results compared to a proportion of 10%. In our analyses, we kept a very similar proportion in both simulated and empirical datasets (56% and 50%). Consequently, according to Marie et al. (2010), our accuracy should not have suffered from low hybrid proportions, but only from low *F*_ST_ values (0.18). It can be expected that for populations with a very similar genetic pool, the discrimination of hybrids might only be possible when the contribution of one parental population becomes significantly greater, as in the case of backcrosses. Nonetheless, it can also quickly become impossible to distinguish backcrosses from parental classes (Marie et al. 2010).

Overall, NEWHYBRIDS seems to be more accurate than STRUCTURE for detecting hybrids and parent individuals with, however, a low discriminative power between hybrid classes. To improve results, the use of a larger number of microsatellites or of additional markers (such as mtDNA, Y chromosome markers, or single nucleotide polymorphism (SNP), Vilà et al. 2003; Koskinen et al. 2004) would be appropriate. However, it is highly probable that the complexity of crossbreeding might add an extra level of difficulty. The H’mong breed is not subjected to any selection scheme, if not the proximity of a member of the opposite sex. If, in addition, overlapping generations have occurred, then the pedigree looks more like a spider's web than a tree. We believe such a pattern to be very common in local breeds in developing countries. In the case of H’mong breed, this complexity can be partly assessed by the substantial diversity of phenotypes found. Coat color phenotypes in pigs, other than black coat, are due to well-known mutations in the E/MC1R and I/KIT genes (Kijas et al. 1998, 2001; Johansson et al. 2005) that were absent from the HG population. Still, Spotted pigs, which phenotypically showed a heritage at one stage of hybridization, were genetically similar to Black pigs, suggesting many generations of backcrossing. Consequently, taking all this into account, a combination of descriptors, phenotypes, and multiple types of genetic markers would improve the estimation of the degree of exotic gene introgression into local breeds but, once again, due to the complexity of their pedigree, this may be inaccurately estimated.

### Conservation consequences of crossbreeding

The justification for conserving the genetic diversity of local breeds is straightforward. Genetic traits that are or may be useful to husbandry are worth conserving, including those that affect disease resistance, environmental tolerance, and taste. However, farmers are more inclined to raise breeds that are popular on the local market and for which economic gain will be the highest. Many studies have shown that the introduction of exotic breeds is mainly driven by economic forces. In Vietnam, almost 80% of pig production is still in the hands of smallholders, but commercial pig production, raising exotic prolific breeds, is growing fast (FAO AGAL 2005). A popular solution for promoting the rearing of local breeds has been to improve their genetic traits by crossbreeding with highly productive exotic breeds. While an improvement in their production traits can be expected, it is not always the case, as for Mong Cai and Bam Vietnamese local breeds (Herold et al. 2010). Moreover, recent concerns have been raised about the genetic swamping of local gene pools by exotic gene pools. Once exotic breeds are introduced, be it controlled or uncontrolled, crossbreeding might lead to genetic erosion by dilution. For instance, uncontrolled crossbreeding was an important factor in the genetic erosion of the local Yucatan Creole pig breed (Drucker and Anderson 2004). While the pure Creole breed accounted for 100% of the pig population in 1940, by 1999 pure Creole pigs amounted to fewer than 100 individuals. Similarly, in the highland areas of Kenya, the use of crosses between exotic cattle breeds with zebu cattle such as the Small East African zebu (SEAZ) has resulted in extensive breed substitution of the SEAZ (Omore et al. 1999).

Many studies on wild species have demonstrated the consequences of genetic dilution by introgressive hybridization, ultimately leading to extinction (Levin et al. 1996; Rhymer and Simberloff 1996). The consequences of introgression may include reduced fitness, population viability, and survival, as well as disruption of local adaptation and life-history characteristics via the introduction of maladaptive gene complexes (Rhymer and Simberloff 1996; Allendorf et al. 2001; McGinnity et al. 2003).

Our results showed that the Vietnamese H’mong pig breed exhibited at least 25% of admixture with exotic breeds due to uncontrolled crossbreeding. According to our model, this exotic introgression will greatly affect the genetic diversity of the local breed by modifying the nature of the genetic diversity within populations and by reducing the level of differentiation between populations. Hansen et al. (2006) showed that in about 50 years, *F*_ST_ between contemporary brown trouts decreased around twofold compared to ancestral samples. While genetic differentiation was still very low between White and Black HG pigs (0.061), it disappeared after 100 generations (*F*_ST_= 0.0002). Conversely, differences in expected heterozygosity (from 0.80 to 0.78) and effective alleles (5 to 4.5) were not so pronounced. The apparent status quo in genetic diversity with exotic introgression has to be taken with caution. Indeed, in our study this was due to the contribution of new alleles from exotic breeds. Similar stability of genetic diversity has been observed in Atlantic salmon with crossbreeding occurring with individuals from multiple farms collectively containing a large genetic variation that mitigates genetic erosion (Bourret et al. 2011).

Private alleles and differences in allele frequency distribution are considered the most significant parameters for population differentiation (Beaumont et al. 2001; Oliveira et al. 2008). According to our results, if management practices are unchanged, nearly 50% of private alleles will be lost within 30 generations, and 100% after 60 generations. If admixture is stopped, only 27% will be lost after 100 generations. While “private alleles” does not automatically mean rare, it is very common that rare alleles are also private. It has been shown that rare alleles can indeed be relevant for some production traits in other livestock species (Grobet et al. 1997; Freking et al. 2002; Smit et al. 2003). The intensity of allelic loss might be considerably variable depending on the level of crossbreeding in the introduced breed. For example, the use of exotic pure individuals from Landrace and Yorkshire breeds, with which 50% of alleles were not shared with Black HG pigs, might considerably accelerate and increase the loss of local alleles. Indeed, Muir et al. (2008) found that at least 50% of genetic diversity in ancestral chicken breeds has been lost in commercial pure chicken lines. By using White HG pigs, which are already hybrids and have 90% of alleles in common with Black HG pigs, householders might have slowed down and limited allelic loss. Speed loss is not only affected by individuals from which introgression occurs but also by the amount of admixture and the rate of mortality which, in a constant population size, would be similar to the recruitment rate. As expected, our simulations showed that an increase in admixture and MRs would lead to an acceleration of allelic loss. The effect of admixture was found to be greater than that of the MR. The direct implication of these results would be that improving sanitary conditions in low-input systems might slow down genetic erosion even when indiscriminate crossbreeding cannot be controlled.

## Conclusions

The originality of our study came from the combination of three types of information: (1) phenotypic observation, (2) householder interviews, and (3) genetic data. Such a global procedure should be recommended to gain precision for characterizing and establishing AnGR conservation policies. Our results suggest major gene flow between exotic breeds and the H’mong pig breed. We expect that future AnGR management and improvement projects will carefully consider the use of exotic genetic pools. Our estimation of the number of generations for homogenization of the introduced and local populations could provide some clues for conservation biologists in wildlife management. Translocated populations may genetically homogenize faster that we thought with local populations and, as a consequence, greatly modify the prior management units and induce the disappearance of local adaptations. In our study, we believe that admixture was underestimated due to tool limitations in the case of closely related populations. Further studies using high-throughput SNP chips available in many species such as the pig (Ramos et al. 2009) might improve detection of hybrid classes and ARs, even if these tools were developed mainly on commercial breeds (Matukumalli et al. 2009; Ramos et al. 2009). In addition, we would advise using private alleles and *F*_ST_ to quantify the degree of genetic erosion.

## Supporting Information - Evolutionary Analysis

**Figure S1.** Estimates of Δ*k* for two datasets: (1) the global dataset including exotic breeds and HG populations; and (2) the Ha Giang population including the three phenotypes (White, Black, and Spotted).

**Figure S2.** Isolation by distance pattern of the Black HG pig population.
