# Supplementary figures and images for: Uncontrolled admixture and loss of genetic diversity in a local Vietnamese pig breed
Source: Ecol Evol. 2012 May;2(5):962–75. doi: 10.1002/ece3.229 (PMC3399162; doi:10.1002/ece3.229)

Exotic &  
HG

HG

$D.\Delta k$

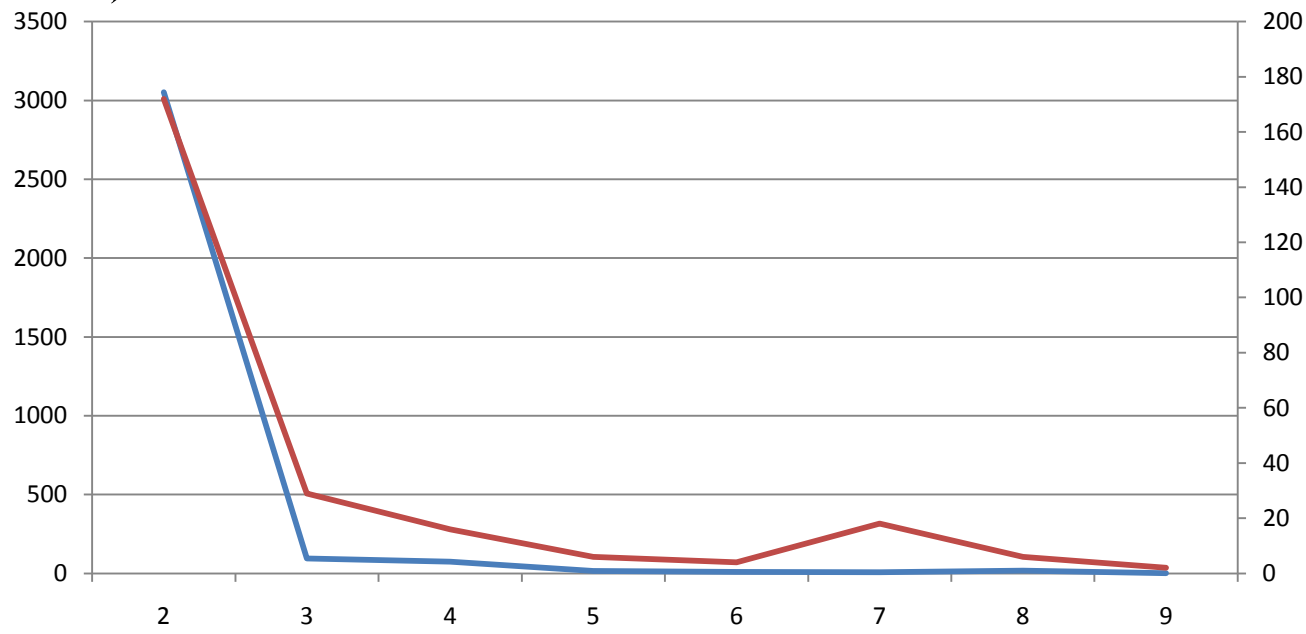

Exotic and HG populations  
HG

k

Supplement: Supplementary file 1 [file ece30002-0962-SD1.pdf]

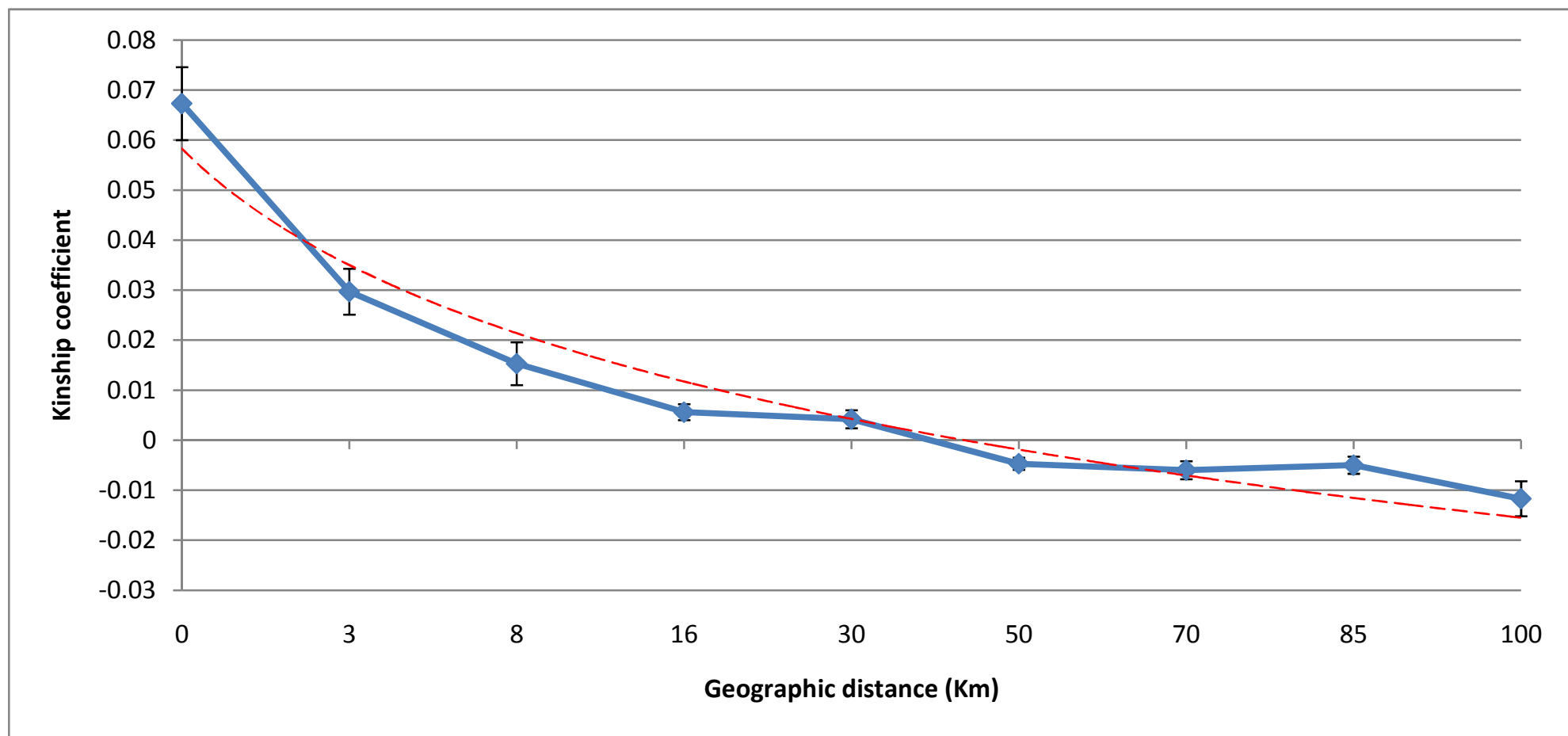

Supplement: Supplementary file 2 [file ece30002-0962-SD2.pdf]
